# Apigenin Attenuates Adriamycin-Induced Cardiomyocyte Apoptosis via the PI3K/AKT/mTOR Pathway

**DOI:** 10.1155/2017/2590676

**Published:** 2017-06-08

**Authors:** Wei Yu, Huirong Sun, Wenliang Zha, Weili Cui, Ling Xu, Qing Min, Jiliang Wu

**Affiliations:** ^1^Department of Pharmacology, Hubei University of Science and Technology, Xianning 437100, China; ^2^Department of Cardiology, Xianning Central Hospital, Xianning 437100, China; ^3^Department of Surgery, Hubei University of Science and Technology, Xianning 437100, China

## Abstract

Treatment with Adriamycin (ADR) is one of the major causes of chemotherapy-induced cardiotoxicity and therefore is the principal limiting factor in the effectiveness of chemotherapy for cancer patients. Apigenin (API) has been shown to play a cardioprotective role. The present study examined the effect of API on ADR-induced cardiotoxicity in mice. Sixty male Kunming mice were randomly divided into 4 groups: a control group, ADR model group, low-dose API treatment group (125 mg·kg^−1^), and high-dose API treatment group (250 mg·kg^−1^). Blood samples were taken to evaluate a spectrum of myocardial enzymes. Cardiomyocyte apoptosis was measured using a TUNEL assay, and cardiomyocyte autophagy was observed using electron microscopy. Moreover, apoptosis-related proteins, such as Bax and Bcl-2, autophagy-related proteins, including Beclin1 and LC3B, and PI3K/AKT/mTOR pathway-related proteins were examined with western blot. Our results demonstrate that ADR caused an increase in the serum levels of cardiac injury markers and enhanced cardiomyocyte apoptosis and autophagy. API administration prevented the effects associated with ADR-induced cardiotoxicity in mice and inhibited ADR-induced apoptosis and autophagy. API also promoted PI3K/AKT/mTOR pathway activity in ADR-treated mice. In conclusion, API may have a protective effect against ADR-induced cardiotoxicity by inhibiting apoptosis and autophagy via activation of the PI3K/AKT/mTOR pathway.

## 1. Introduction

Since the introduction of Adriamycin (ADR) in the late 1960s, it has been widely applied to the treatment of various types of malignancies, including breast cancer, multiple myeloma, neuroblastoma, leukaemia, and sarcomas, and it is associated with satisfactory clinical effects [1, 2]. However, the use of ADR has been limited due to its severe cardiotoxic side effect, which causes fatal congestive heart failure [3]. ADR-induced cardiotoxicity includes acute and chronic cardiotoxicity. Acute cardiotoxicity occurs during or immediately after ADR treatment and is characterized by aberrant electrophysiology and arrhythmias [4]. Chronic cardiotoxicity may occur weeks or many years after ADR administration and presents as cardiac dysfunction [5]. Since it was first revealed that ADR was highly cardiotoxic, numerous studies have been devoted to determining its underlying mechanisms, yet the aetiology of ADR-induced myocardial injury is not fully clear. To date, several cellular mechanisms, including myocardial fibrosis, apoptosis, oxidative stress, mitochondrial dysfunction, and autophagy, have been proposed to account for the cardiomyopathy caused by ADR [6, 7]. To minimize and manage ADR-associated cardiotoxicity, several effective approaches have been put forward, such as dosage optimization or combination therapy, and many of these strategies still await verification in large scale clinical trials [8].

Apigenin (API), a natural flavonoid, possesses a broad spectrum of biological properties, including antioxidative, anti-inflammatory, anticancer, and neuroprotective effects [9]. It has also been demonstrated to be associated with reduced risks of cardiovascular diseases, such as experimental autoimmune myocarditis [10], lipopolysaccharide-induced heart injury [11], and ischaemia/reperfusion (I/R) injury [12, 13]. Studies have also reported that API plays a cytoprotective role against ADR-induced cardiotoxicity [14]. However, the mechanism has not been fully clarified.

Therefore, the objective of this study was to examine the effects of API on ADR-induced cardiomyopathy using a mouse model of ADR cardiotoxicity. We tested the hypothesis that API attenuates myocardial apoptosis and regulates autophagy induced by ADR and explored whether the effects of API are mediated through the PI3K/AKT/mTOR pathway.

## 2. Materials and Methods

### 2.1. Experimental Animals

Sixty healthy Kunming mice (26 ± 2 g) were purchased from the Laboratory Animal Center of the Preventive Medicine Academy of Hubei province. Before starting the experiments, the mice were adapted to the laboratory conditions for seven days. The experimental animals were housed with a 12 h light/dark cycle at a temperature of 23~25°C and humidity of 55~60%. The protocols used in this study conformed to the Guide for the Care and Use of Laboratory Animals published by the National Institutes of Health (NIH Publication 85-23, revised 1996). All experiments were approved by the Committee of Experimental Animals of Hubei University of Science and Technology.

### 2.2. Experimental Groups and Management

Animals were randomly assigned into two groups: a control group (*n* = 15) and an ADR group (*n* = 45). The ADR group was divided into three subgroups: ADR only without API (ADR, *n* = 15), low-dose API (Low-API, 125 mg/kg/day, *n* = 15), and high-dose API (High-API, 250 mg/kg/day, *n* = 15). ADR (3 mg/kg/day; Zhejiang Hisun Pharmaceutical Co. Ltd., China) was injected intraperitoneally into animals at an interval of 48 h (in total, eight times at a cumulative dose of 24 mg/kg). The mice in the control group received injections of 0.9% sterile saline.

API (Shijiazhuang City Lvchuan Biotechnology Co. Ltd., China) was mixed with 0.5% sodium carboxymethyl cellulose (CMC-Na) to form a suspension. All API-treated groups were treated daily via gastric gavage for seventeen days with a 125 or 250 mg/kg/day dose as described above. The ADR-only and control (NC) groups were treated with vehicle (CMC-Na) only. At the end of experiment, three mice died in ADR group and three and one mice died in low-dose and high-dose apigenin treatment group, respectively. These mice all died due to cardiomyotoxicity.

On the 17th day after the first treatment, the mice were sacrificed, and blood samples were collected. A number of hearts were fixed with 2.5% glutaraldehyde fixative for electron microscopy analysis, and the others were stored at −80°C for western blot analysis.

### 2.3. Electron Microscopy Analysis

Approximately 1 mm^3^ of tissue was removed from the left ventricle and immediately fixed in 2.5% glutaraldehyde for 2 h. Following fixation in 1% osmium tetroxide solution, the samples were rinsed in phosphate-buffered saline (0.1 mol/L, pH 7.4). Subsequently, the tissues were dehydrated in an ascending alcohol series. The tissues were then embedded in Epon. Finally, the tissues were cut on an ultramicrotome and double-stained with uranyl acetate and lead citrate. The samples were examined using a transmission electron microscope (FEI TecnaoG^2^12) and the number of autophagic vacuoles per field within a cardiomyocyte was calculated.

### 2.4. Measurement of Myocardial Enzymes

Serum aspartate amino transferase (AST), lactate dehydrogenase (LDH), and creatine kinase (CK) were measured to assess myocardial injury. These myocardial enzymes were measured using kits purchased from Nanjing Jiancheng Institute of Biological Engineering. The detailed procedures were performed according to the manufacturer's instructions for the different reagent kits.

### 2.5. Terminal Deoxynucleotidyl Transferase dUTP Nick End Labelling Assay

Apoptosis in the heart was identified using a terminal deoxynucleotidyl transferase dUTP nick end labelling (TUNEL) kit (Roche Applied Science, USA). Paraffin-embedded sections of heart tissues were deparaffinized with xylene and rehydrated in a graded ethanol series. The sections were incubated with proteinase K at 37°C for 15 min and then incubated with a reaction mixture of terminal deoxynucleotidyl transferase at 4°C overnight. Finally, 3,3′-diaminobenzidine tetrahydrochloride (DAB) was added to stain nuclei, and images were captured with a fluorescence microscope (Olympus BX53, Japan). The apoptotic index was estimated using the formula: TUNEL-positive cells/total cells × 100%.

### 2.6. Western Blot Analysis

For the western blot analysis, hearts were lysed in RIPA buffer, 1 mM Na_3_VO_4_, 1 mM phenylmethylsulfonyl fluoride (PMSF), and protease inhibitors and then centrifuged at 12,000 rpm for 15 min at 4°C. Protein concentrations were determined with a bicinchoninic acid (BCA) protein assay (Beyotime, China). Aliquots of total heart extract (30–40 *µ*g) were loaded onto SDS-PAGE gels of different concentrations and then transferred to PVDF membranes. The membranes were blocked in 5% nonfat dry milk solution in a triethanolamine-buffered saline with Tween 20 (TBST) and then incubated with commercial antibodies specific for Bax, Bcl-2, Beclin1, LC3, GAPDH (CST, CA, USA), p-mTOR, mTOR, p-AKT (s473), AKT1/2/3, and PI3K p85 (Abcam, CA, USA) at 4°C overnight. On the second day, the membranes were washed in TBST three times. Then, the membranes were incubated with horseradish peroxidase-conjugated secondary antibodies at a 1 : 5000 dilution at room temperature for 1 h. After incubation in an ECL (Pierce Biosciences, USA) solution, membranes were exposed to Syngene Gene Genius. The intensity of protein bands was semiquantified using image analysis software.

### 2.7. Data Analysis

Statistical analysis was performed using SPSS 11.5 software. Data are presented as the mean ± SEM. The data were analysed using one-way ANOVA with LSD post hoc test. Differences were considered significant if *P* < 0.05.

## 3. Results

### 3.1. Apigenin Alleviated ADR-Induced Myocardial Injury

**Figure 1:**
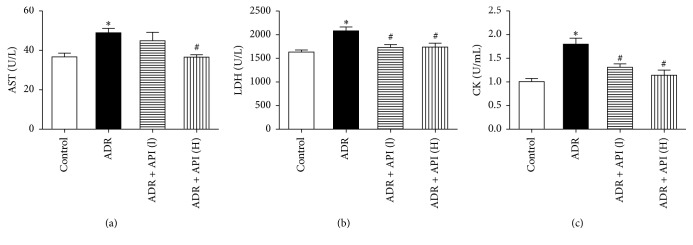
Apigenin alleviated ADR-induced myocardial injury. (a) Apigenin inhibited serum AST release. (b) Apigenin reduced serum LDH release. (c) Apigenin reduced serum CK contents. AST: aspartate amino transferase; LDH: lactate dehydrogenase; CK: creatine kinase-MB; control: control group; ADR: ADR model group (24 mg/kg); ADR + API (L): low-dose apigenin treatment group (125 mg/kg); ADR + API (H): high-dose apigenin treatment group (250 mg/kg); *n* = 10 per group. Values are presented as the mean ± SEM. ^*∗*^*P* < 0.05 compared with the control group; ^#^*P* < 0.05 compared with the ADR model group.

The serum concentrations of AST, LDH, and CK are important biochemical markers of myocardial injury. As shown in Figure 1, compared to the control group, the ADR group showed significant increases in serum myocardial enzyme levels (relative to the control group, AST, *P* = 0.003; LDH and CK, *P* < 0.001), whereas API significantly decreased serum enzyme levels in a dose-dependent manner. Treatment with 250 mg/kg API significantly decreased AST release (*P* = 0.003). In addition, API at 125 and 250 mg/kg markedly inhibited LDH and CK release into the serum (relative to the ADR group, for LDH in the API 125 and 250 mg/kg groups, *P* = 0.001 and 0.002, resp.; for CK in the API 125 and 250 mg/kg groups, *P* = 0.001 and *P* < 0.001, resp.), which indicates that these doses of API can protect against ADR-induced myocardial injury.

### 3.2. Apigenin Inhibited ADR-Induced Cardiomyocyte Apoptosis

**Figure 2:**
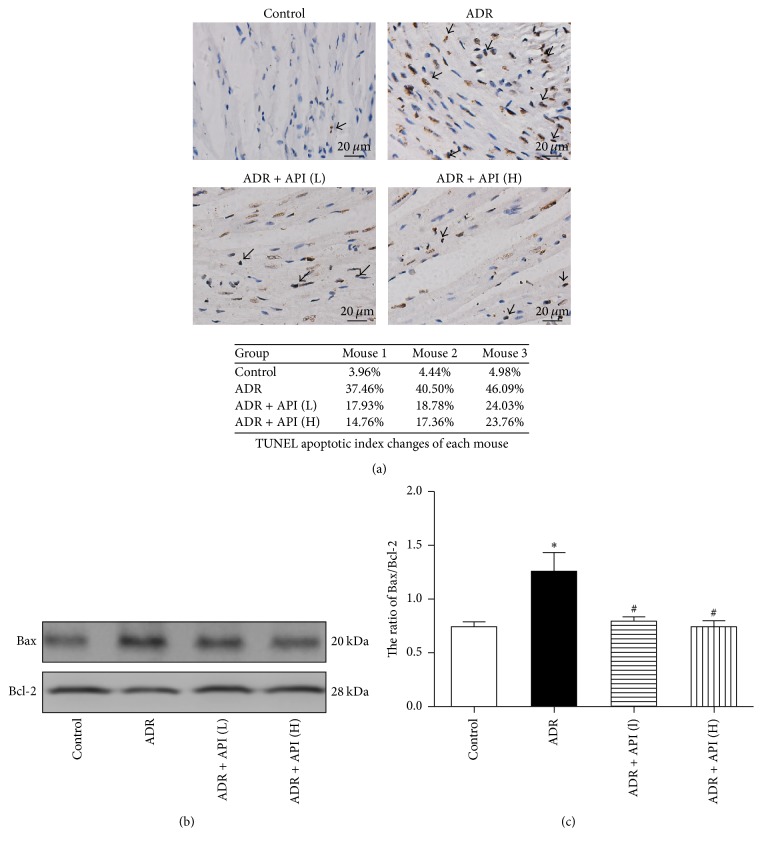
Effect of apigenin on apoptosis and related protein expression in hearts from ADR-treated mice. (a) Representative pictures of TUNEL assay results (magnification: 400x) and TUNEL apoptotic index were determined by calculating the ratio of TUNEL-positive cells to total cells. Arrows indicate positive cells; *n* = 3 per group. (b) Representative pictures of Bcl-2 and Bax expression; *n* = 5 per group. (c) Quantitative analysis of Bax/Bcl-2 ratios; *n* = 5 per group. Values are presented as the mean ± SEM. ^*∗*^*P* < 0.05 compared with the control group; ^#^*P* < 0.05 compared with the ADR model group.

Because apoptosis is a common cell-death pathway, we examined apoptosis using a TUNEL assay. As shown in Figure 2(a), the number of TUNEL-positive cells was much higher in the ADR group than in the control group. After treatment with API, the number of apoptotic cells was remarkably downregulated. Moreover, to verify whether API protected against apoptosis, proteins such as Bax (a proapoptotic protein) and Bcl-2 (an antiapoptotic protein) were assessed in all samples. As shown in Figure 2(b), the Bax/Bcl-2 ratio, an indicator that is typically considered to represent the susceptibility of cells to induced apoptosis, was markedly increased in the ADR model group (*P* < 0.001 versus the control group). After treatment with 125 and 250 mg/kg API, the upregulation trend of the Bax/Bcl-2 ratio was significantly reduced (*P* = 0.007 and 0.001, resp., versus the ADR group).

### 3.3. Apigenin Inhibited ADR-Induced Cardiomyocyte Autophagy

**Figure 3:**
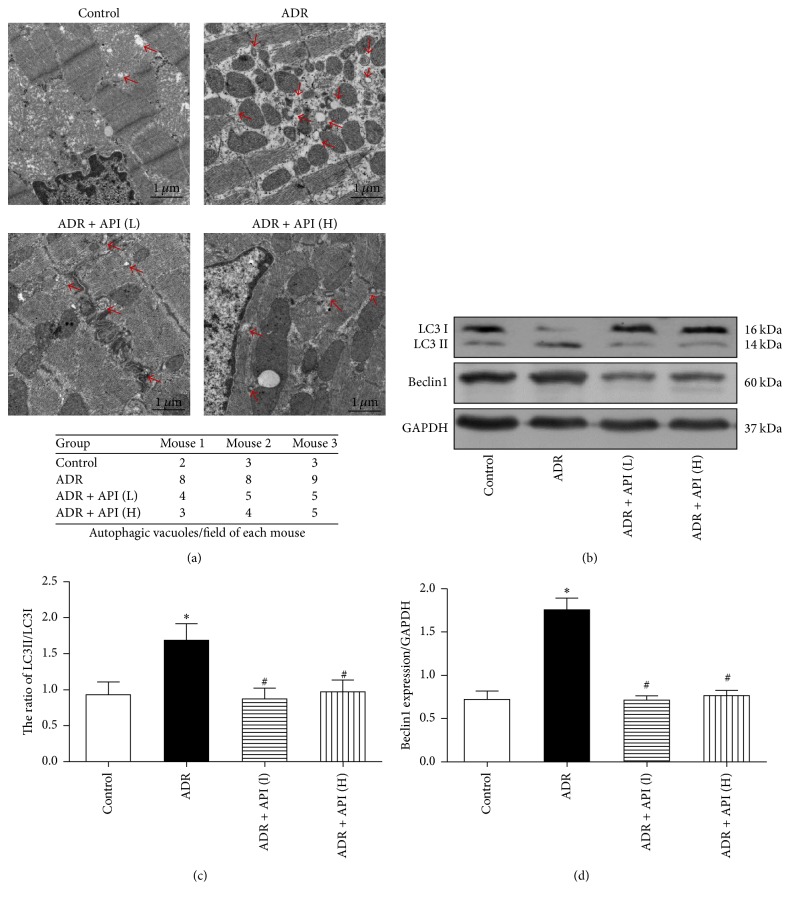
Effect of apigenin on autophagy and related protein expression in hearts from ADR-treated mice. (a) Representative transmission electron micrographs of left ventricular specimens. Arrows indicate autophagic vacuoles; *n* = 3 per group. (b) Representative pictures of Beclin1 and LC3B expression; *n* = 5 per group. GAPDH served as the loading control. (c) Quantitative analysis of LC3BII/LC3B I ratios; GAPDH served as the loading control; *n* = 5 per group. (d) Beclin1 expression levels were quantified via densitometry and normalized to the expression of GAPDH, which was used as a loading control; *n* = 5 per group. Values are presented as the mean ± SEM. ^*∗*^*P* < 0.05 compared with the control group; ^#^*P* < 0.05 compared with the ADR model group.

Previously, we observed cardiomyocyte autophagy using electron microscopy. Autophagic vacuoles containing subcellular materials were observed in the ADR group, and the number of autophagic vacuoles was decreased after treatment with API (Figure 3(a)).

Moreover, we determined the effect of API on the expression of autophagy marker proteins, including Beclin1 and LC3B, with western blot. Beclin1 plays a key role in autophagosome formation. The amount of LC3B II/I is reported to be proportional to the number of autophagic vacuoles. As shown in Figure 3(b), the expression of Beclin1 and LC3B II/I was increased in hearts from ADR-treated mice (for Beclin1 and LC3B II/I, *P* = 0.003 and *P* = 0.01 versus the control group). More interestingly, API treatment significantly inhibited Beclin1 and LC3B II/I expression (relative to the ADR group, for Beclin1 in the API 125 and 250 mg/kg groups, *P* = 0.003 and 0.001, resp.; for LC3B II/I in the API 125 and 250 mg/kg groups, *P* = 0.006 and 0.013, resp.), suggesting that API protects against ADR-induced cardiotoxicity by inhibiting autophagy.

### 3.4. Apigenin Activated the PI3K/AKT/mTOR Pathway in ADR-Treated Mice

**Figure 4:**
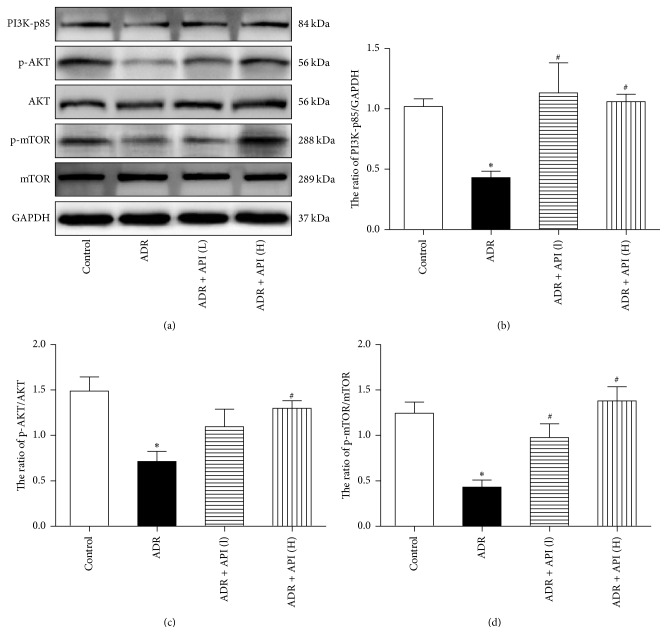
Effect of apigenin on the PI3K/AKT/mTOR pathway in hearts from ADR-treated mice. (a) Representative pictures of PI3K-p85, p-AKT, AKT, p-mTOR, and mTOR expression. GAPDH served as the loading control; *n* = 5 per group. (b) Quantitative analysis of the PI3K-p85/GAPDH ratio; expression levels were quantified via densitometry and normalized to the expression of GAPDH, which was used as a loading control; *n* = 5 per group. (c) Quantitative analysis of the p-AKT/AKT ratio; GAPDH served as the loading control; *n* = 5 per group. (d) Quantitative analysis of the p-mTOR/mTOR ratio; *n* = 5 per group. GAPDH served as the loading control. Values are presented as the mean ± SEM. ^*∗*^*P* < 0.05 compared with the control group; ^#^*P* < 0.05 compared with the ADR model group.

The PI3K/AKT/mTOR pathway plays important roles in cell apoptosis and autophagy. To explore whether the effects of API on cell apoptosis and autophagy in ADR-treated mice are related to this pathway, we examined the signalling proteins in the pathway. As shown in Figure 4, compared with levels in control mice, the expression of p85, the catalytic subunit of PI3K, and the phosphorylation of AKT and mTOR were downregulated in ADR-treated mice (PI3K *P* = 0.007, p-AKT/AKT *P* = 0.002, and p-mTOR/mTOR *P* < 0.001 versus the control group). However, the ADR-induced PI3K/AKT/mTOR inactivation was recovered by treatment with API (relative to the ADR model group, for PI3K in the API 125 and 250 mg/kg groups, *P* = 0.002 and 0.005, resp.; for p-AKT/AKT in the API 125 and 250 mg/kg groups, *P* = 0.078 and 0.011, resp.; and for p-mTOR/mTOR in the API 125 and 250 mg/kg groups, *P* = 0.009 and <0.001, resp.). Therefore, the western blotting results revealed that API significantly enhanced the PI3K/AKT/mTOR pathway.

## 4. Discussion

ADR, an anthracycline, is one of the most commonly used anticancer drugs. However, the cardiotoxicity of ADR presents a major limiting factor for its wide therapeutic application in oncology. Previous studies have demonstrated that cardiotoxicity can be induced by 15–25 mg/kg ADR [15, 16]. Researchers also found that 24 mg/kg ADR and higher cumulative doses can lead to severe cardiac lesions in mice [17]. It has been reported that a deficiency in oxygen or glucose supply may damage cardiac muscle cell membranes and eventually cause the leakage of LDH, CK, and AST from heart mitochondria, and, therefore, these serum enzymes are considered the best cardiac injury biomarkers, whose increase indicates cardiac deterioration. In this study, cardiotoxicity caused by a dose of 24 mg/kg ADR manifested biochemically as an increase in these three cardiac enzymes, and these outcomes were in keeping with previous results reported by other researchers [18, 19]. We found that API remarkably alleviated the release of these enzyme markers especially when administered at a dose of 250 mg/kg. These results suggest that the protective role of API may be due to the inhibition of myocardial damage, thereby restricting the leakage of these enzymes.

Previous studies have confirmed that ADR-induced cardiotoxicity can be attributed to multiple mechanisms, but increased myocardial cell apoptosis is the most widely accepted mechanism [20]. Given that ADR triggers apoptosis in a large number of myocardiocytes, which causes dilated cardiomyopathy and heart failure, a trend of myocardial cell-death inhibition was noticed in ADR cardiotoxicity treatments. Accumulating lines of evidence have shown that API shows antitumour activity and induces apoptosis in many cancer cell lines in a dose-dependent manner [21, 22]. Conversely, API also acts as an antiapoptotic agent, providing cardioprotection through suppression of myocardial apoptosis [11]. Our data revealed that ADR increased heart nuclear TUNEL staining, which is as an index of cardiac cell apoptosis, and API significantly abrogated the cardiomyocyte apoptosis induced by ADR. Furthermore, mitochondrial dysfunction has been recently highlighted as a major event associated with ADR-induced cardiomyocyte apoptosis [23]. Moreover, proapoptotic Bax and antiapoptotic Bcl-2 proteins are proposed to play critical roles in deciding cell survival or death [24]. Thus, we examined the effects of API on the protein expression of Bax and Bcl-2. The ratio of Bax/Bcl-2 was found to be increased in the ADR treatment group. However, these proapoptotic effects were reversed by API. Taken together, these results suggest that API may protect cardiomyocytes against ADR by suppressing apoptosis.

Autophagy is an evolutionarily conserved self-digestion process in which long-lived proteins and organelles are sequestered within cytosolic double-membraned vesicles, named autophagosomes, and end up in lysosomes for degradation and recycling. Autophagy has been viewed as another type of cell death, in addition to apoptosis, and is recognized as a double-edged sword. Recent evidence suggests that the dysregulation of cardiomyocyte autophagy may play a critical role in ADR-induced cardiotoxicity. However, the role of autophagy in ADR-induced cardiotoxicity is still controversial. Most studies have demonstrated that ADR increases autophagy signalling, and, in several in vivo and in vitro studies, inhibiting autophagy was sufficient to attenuate ADR-induced cardiotoxicity [25–27]. In the current study, we utilized various methods to monitor changes in autophagy. Electron microscopy images revealed that the formation of autophagic vacuoles was increased in hearts from ADR-treated mice, which was consistent with most of the current evidence. Beclin1 is part of class III PI3K protein complex and serves as a platform to recruit multiple autophagy-related proteins to the isolation membrane. Interestingly, emerging evidence has indicated that cross-talk exists between autophagic and apoptotic pathways. For example, the antiapoptotic protein Bcl-2 inhibits autophagy by binding and sequestering Beclin1 away from the class III PI3K complex and eventually suppresses Beclin1-mediated autophagy [28]. Conversion of cytosolic LC3 I into autophagosome-bound LC3 II is a well-established indicator for autophagosome formation. Here, we found that ADR treatment significantly increased the levels of both the ratio of LC3B II/I and Beclin1, which was consistent with the electron microscopy results, implying that autophagy might be a key target of ADR. Studies have also reported that API can regulate autophagy in cancer cells, but this is still controversial. Some researchers found that API induced autophagy in cancer cells such as TF1 erythroleukaemia cells and breast cancer T47D and MDA-MB-231 cells [29]. However, another research reported that API inhibited autophagy in serum-starved SH-SY5Y cells [30]. Thus, cancer cells are different from normal tissue cells, and API plays a different role in autophagy in different cell types. Interestingly, we found that when mice were coexposed to ADR and API, autophagosome production and the ratio of LC3B II/I and Beclin1 expression were downregulated. Thus, API might recover the autophagy dysfunction induced by ADR.

The PI3K-AKT-mTOR pathway is required for the prosurvival signalling cascade under a wide variety of circumstances. In previous studies, it was revealed that ADR induced myocardial apoptosis by downregulating the PI3K/AKT/mTOR survival pathway [20]. Additionally, the PI3K/AKT/mTOR pathway is confirmed to be a negative regulator of autophagy via several downstream targets [31]. The class III PI3K inhibitor 3-MA and other approaches such as ghrelin, a metabolic regulatory peptide, have been found to suppress ADR-induced autophagy and attenuate cardiomyocyte apoptosis [7, 32]. Pisonero-Vaquero S reports that API can protect HK-2 cells against cisplatin cytotoxicity by stimulating the PI3K/AKT pathway [33]. As a result, it would be intriguing to know whether manipulation of the PI3K/AKT/mTOR pathway by API contributes to its cardioprotective effects against ADR. Our results show that API treatment upregulated the phosphorylation of AKT and mTOR, as well as the expression of PI3K. Therefore, the API-mediated reduction of ADR-induced apoptosis and autophagy might be attributed to the activation of PI3K downstream effectors, such as AKT and mTOR.

Taken together, these results show that API exerts a beneficial effect against ADR-induced cardiotoxicity, which may involve the alleviation of apoptosis and autophagic flux in cardiomyocytes through PI3k/AKT/mTOR pathway modulation.
